# Limitation of Number of Strains and Persistence of False Positive Loci in QTL Mapping Using Recombinant Inbred Strains

**DOI:** 10.1371/journal.pone.0102307

**Published:** 2014-07-17

**Authors:** Lishi Wang, Yan Jiao, Yanhong Cao, Gaifen Liu, Yongjun Wang, Weikuan Gu

**Affiliations:** 1 Department of Orthopedic Surgery and BME, Campbell-Clinic, University of Tennessee Health Science Center, Memphis, Tennessee, United States of America; 2 Mudanjiang Medical College, Mudanjiang, PR China; 3 Institute of Kaschin-Beck Disease, Center for Endemic Disease Control, Chinese Center for Disease Control and Prevention, Harbin Medical University; Key Laboratory of Etiologic Epidemiology, Education Bureau of Heilongjiang Province and Ministry of Health (23618104), Harbin, China; 4 Department. of Neurology, Beijing Tiantan Hospital, Capital Medical University, Beijing, China; 5 Department of Basic Medical Research, Inner Mongolia Medical University, Inner Mongolia, China; University of Illinois at Chicago, United States of America

## Abstract

While the identification of causal genes of quantitative trait loci (QTL) remains a difficult problem in the post-genome era, the number of QTL continues to accumulate, mainly identified using the recombinant inbred (RI) strains. Over the last decade, hundreds of publications have reported nearly a thousand QTL identified from RI strains. We hypothesized that the inaccuracy of most of these QTL makes it difficult to identify causal genes. Using data from RI strains derived from C57BL/6J (B6) X DBA/2J (D2), we tested the possibility of detection of reliable QTL with different numbers of strains in the same trait in five different traits. Our results indicated that studies using RI strains of less than 30 in general have a higher probability of failing to detect reliable QTL. Errors in many studies could include false positive loci, switches between QTL with small and major effects, and missing the real major loci. The similar data was obtained from a RI strain population derived from a different pair of parents and a RI strain population of rat. Thus, thousands of reported QTL from studies of RI strains may need to be double-checked for accuracy before proceeding to causal gene identification.

## Introduction

Different approaches have been tried to improve and simplify the QTL mapping and candidate selection. Because of the heterozygosity of the individuals and the environmental influences on the phenotype, genetic mapping of QTL using F2 population requires a large population. Sample size limitation in genetic mapping with F2 population is well known. However, sample size limitation for genetic mapping with homozygous individuals has been under debated for the decades. A typical example is the use of standard inbred mouse strains [1]. It was thought that because of the homozygousity, inbred strains are not suffered by the statistic power limitation such as that in F2 segregation populations. However, ever since its starts, in Silico genome-wide mapping of mouse QTL using inbred strains has been a point of debate [2]–[3]. Recent evidence indicated that such association mapping in the population of inbred mouse strains is characterized by a high false-positive rate [4]. It is well known that a significant difference exists between recombinant inbred (RI) strains and available standard inbred strains currently in the Jackson laboratory (http://www.jax.org/) and other mouse resource centers. Both phenotypic traits and genomic regions of RI strains can be tracked down to one of the two parental strains. This feature of the RI strains enables the clear connection between phenotype and genotype association. Therefore, Using RI strains is considered as an easier and more cost-effective approach compared to congenic strains for fine mapping of quantitative trait loci (QTL) identified by F2 designs. The chromosomal content of any such RI strain forms a mosaic of blocks, each alternatively inherited identically by descent from one of the two parents. Because of the homozygousity of RI strain and the ability of repeat with multiple mice from the same RI strain, the effect of most QTL is measurable. The genomic background of the QTL is much less complex than that in F2 population or unrelated inbred strain panels. In spite of these advantages, the number of RI strains is still much less than that of F2 population. A critical question is whether the number of strains is a factor of limitation for the QTL identification by using RI strains. In 2005, Ioannidis used statistic simulation showing that for most study designs and settings, it is more likely for a research claim to be false than true [5] because of the sample sizes and experimental variations. While it received much attention, Goodman and Greenland [6] pointed out that the model employed in the paper constitutes a “proof” that most published medical research claims are false.

In recent years, there has been an increase of mapping with recombinant inbred (RI) strains [7], particularly the RI strains derived from C57BL/6J (B6) X DBA/2J (D2), known as the BXD RI strains [8]–[9]. Although their genomic background of QTL is much less complex than that in the F2 population or unrelated standard inbred strain panels, the number of the RI strains is still much less than that of the F2 population. Problems in sample size and variation in design have been debated. However, no conclusion is reached [5]–[6]. A recently emerging RI resource is the RI strains of Collaborative Cross (CC). CC RI strains derived from eight diverse founder strains, including five classical inbred strains and three wild-derived strains [10]–[11]. The large genetic diversity and high rate of recombination in these RI strains provide a next generation resource for precision mapping of QTL. Therefore, a definitive answer on whether sample size limitation applies to RI strains is critical to the utilization of these RI strains.

What accompanies the debate is the continued accumulation of a large number of QTL across every chromosome and the lack of identification of causal genes of most of the chromosomes. Accordingly, understanding of the size limitation of mouse RI strains will clarify the importance and usefulness of these identified QTL loci. It also helps to find out whether the reported QTL are true genetic loci or the false positive ones. We hypothesized that the reason for not being able to identify the causal genes for those QTL is that most QTL are not true; the identification is either a false positive result or the QTL have a minimal effect on the traits because of the size limitation of the RI strains. The aim of this publication is to investigate the reliability of previously reported QTL with relatively small number of RI strains.

Because of the existence of multiple data collected at different times and/or by different investigators for the variety of phenotypes and availability of genotypes of those RI strains in the GeneNetwork (http://www.genenetwork.org/webqtl/main.py), we were able to collect multiple sets of data to experimentally test the repeatability of RI mapped data.

## Materials and Methods

### Number of strains testing

To test the minimal number of RI strains required to produce the reproducible QTL, we conducted a series of analysis with data of TNFα cytokine expression levels measured two days after infection with H5N1 influenza A virus (GeneNetwork ID: 12971; [12]) of 43 BXD strains, two parental strains, and an F1. For each number of strains reduction, we had five replicates. The number of reduction of strains began with 2 in each cycle after the first reduction. The reduction continued until the number of strains r was reduced to 21.

We used the first set of data of a study model of host factors in the context of influenza virus infection, in which the authors determined (ID 12971) the TNFα cytokine expression level two days after infection with H5N1 influenza A virus [12]. For each number reduction, we had five replicates. The number of reduction of strains began with 2 in each cycle after the first reduction. Thus the testing number of strains in each replicate in the first cycle was 45. For each following test cycle, two different strains sequentially were eliminated from the testing strains. The reduction continued until the number of strains was reduced to 21.

The second data set was from the cerebral cortex volume, adjusted for shrinkage, age, sex, plane of section, and BXD group/epoch [13]. The sample included a set of 56 BXD RI strains (223 animals) (ID 10997). The authors reported the identification of two QTLs–one on chromosome (Chr) 6 at 88+/−5 Mb and another at Chr 11 (41+/−8 Mb).

We eliminated the strains with a randomized method. Each time, we reduced the number of trains to 30, one time for one strain, with 5 replicates.

The third test included four sets of data. Those data have been published and stored in GeneNetwork for public uses. Two sets of data are the deoxycorticosterone (DOC) levels in plasma and cerebral cortex [14]. The authors mapped QTL for both basal cerebral cortical (ID 12569) and plasma DOC (ID 12567) levels. The third set of data was for the virus survival time (ID 10866) [13]. The last set of data was from a study of cocaine response (ID 11485; [15]). For each of those sets, we sequentially reduced the number of strains to 35, 33, 31, and 29, with five replicates for each number of strains.

### Evaluation of detection probability of QTL

To evaluate the probability of detection for each of the three QTL with different numbers of RI strains, we calculated the percentage of detection of each of three QTL at different numbers of RI strains. We examined the marker regression of each of three QTL in each replicate at each reduction number. For each QTL, we used the highest LRS score in our evaluation whenever a QTL is detectable; thus, the LRS score is at the suggestive level. The score was recorded as 0 if a QTL was not at the detectable level.

The probability of QTL detection was calculated using the formula Pd = ∑(hLRS/tLRS)/5, where hLRS is the highest LRS score of the QTL that is above the threshold of suggestive, significant, or highly significant; the tLRS is the threshold of suggestive, significant, or highly significant of the QTL.

#### Comparative testing with samples from RI strains derived from a different cross and from the rat

To explore if there is the limitation for the number of RI strains in QTL mapping, we tested additional two sets of samples. One set of data from RI strain was derived from a reciprocal cross between A/J (A) and C57BL/6J. We tested the number of strains limitation using data of Lung tumor susceptibility (ID: 10070) [16]. The total number of strains using for this phenotype is 44. For the test, we reduced the number of strains into 30, 28, and 26. The other set of data is from the rat model for placental weights (g) in left horn of the uterus (ID10100) [17]. This set of RI strains contains 26 rat RI strains derived from a cross between the spontaneously hypertensive rat (SHR/OlaIpcv = H) and Brown Norway (BN.Lx/Cub or BN = B). We suspected that at least the QTL detected at suggestive level from these strains are not reliable. We therefore conducted two tests with the reductions of two and four strains, each with five replicates.

### Bioinformatics and QTL Analysis

All the analyses of QTL and marker regression were conducted at GeneNetwork (http://www.genenetwork.org/webqtl/main.py).

## Results

### Minimum number of RI strains for precisely detection of QTL for TNFα cytokine expression levels

**Figure 1:**
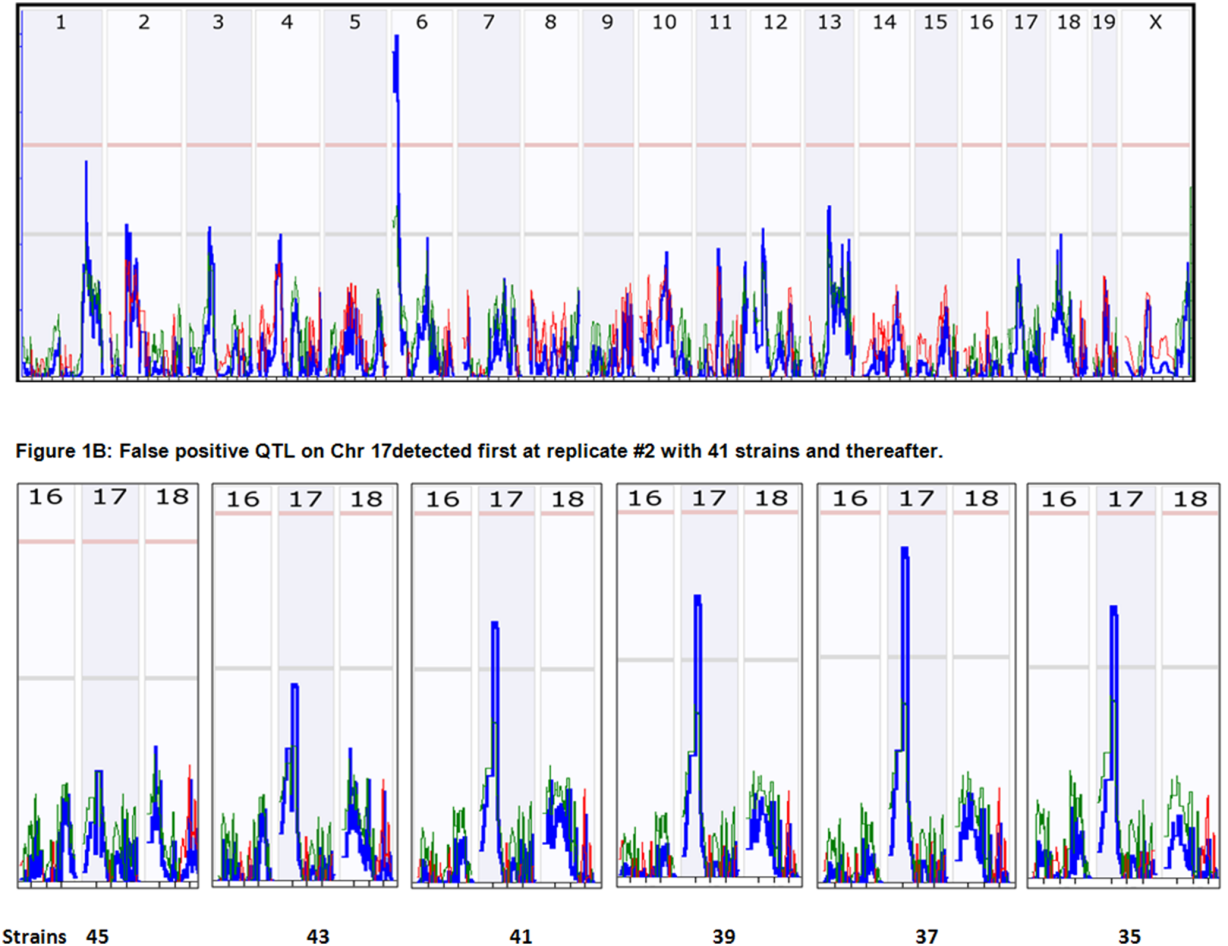
Detection of QTL for TNFα cytokine expression levels using RI strains. The numbers on top of each figure indicate the number of chromosome. Numbers on left vertical bar indicate the LRS values. Pink color lines on the top indicate the threshold for significant level. Light grey lines indicate the threshold for suggestive level. Figure 1A: QTL detected from 46 strains. Figure 1B: False positive QTL on Chr 17 detected first at replicate #2 with 41 strains and thereafter.

With 2000 permutation tests, criteria for the mapping with the original data of total of 46 strains indicted the suggestive LRS = 10.77, significant LRS = 17.20, and highly significant LRS = 20.59. LRS scores of eight QTL loci reached or were higher than the suggestive level (Figure 1A). The major QTL was detected to be on Chr 6, which has the highest LRS score of 25.945. The second locus was on Chr1 with an LRS score of 16.329, and the third locus was on Chr13 with an LRS of 12.927. The other four loci with LRS score at the suggestive level were located on Chr 2, 3, 4, 12, and 18. We then tested the reproducibility or probability of QTL detection (Pd) by sequentially reducing the number of strains (not from the parental strains and the F1).

To evaluate the probability of detection for each of the three QTL loci with different numbers of RI strains, we calculated the percentage of detection of each of three QTL at different numbers of RI strains. We examined the marker regression of each of three QTL in each replicate at each reduction number.

**Figure 2:**
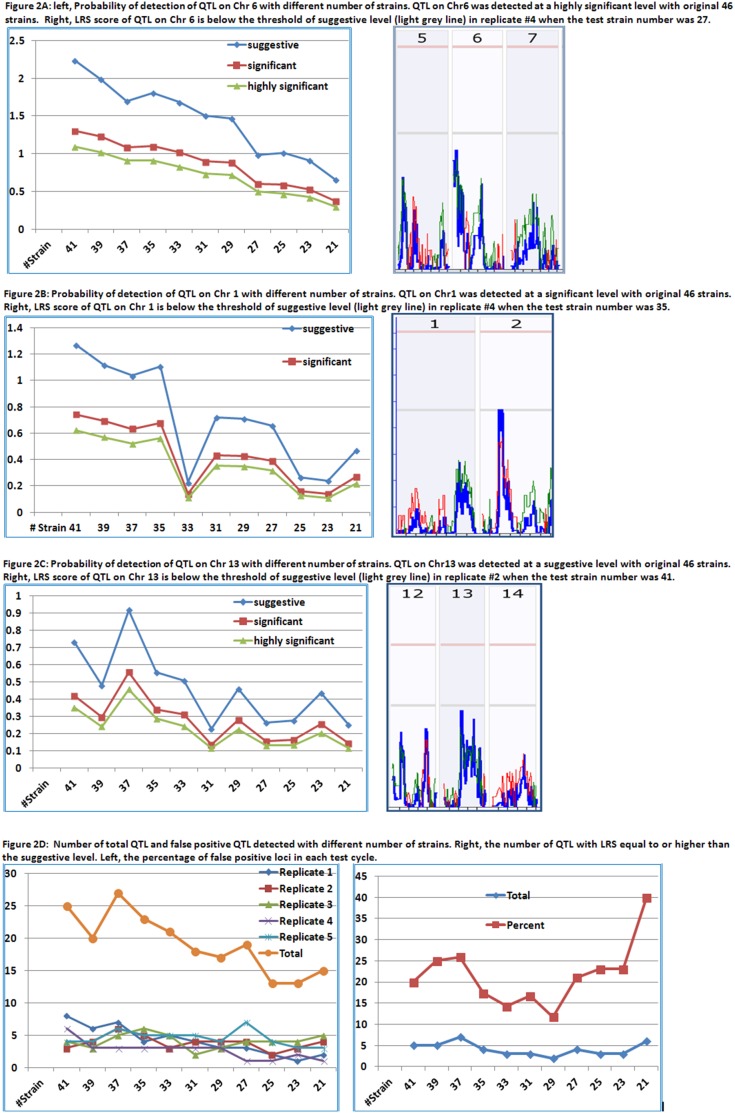
Probability of detection of QTL of TNFα cytokine expression levels with different number of strains. Y-axis indicates the Probability of QTL detection (Pd). X-axis indicates the number of RI strains in the test of Pd. Figure 2A: Pd of QTL on Chr 6 with different number of strains. QTL on Chr6 was detected at a highly significant level with original 46 strains. Figure 2B: Pd of QTL on Chr 1 with different number of strains. QTL on Chr1 was detected at a significant level with original 46 strains. Figure 2C: Pd of QTL on Chr 13 with different number of strains. QTL on Chr13 was detected at a suggestive level with original 46 strains. Figure 2D. Rate of false positive QTL on Chr 17 as a major QTL detected from tests of different number of strains.

For the major QTL on Chr 6, the results indicated that all the tests reproduced the QTL to be on Chr 6 until the number of strains was reduced to 29. The QTL on Chr 6 was rapidly diminished when the numbers of strains were reduced to 29 and 27 (Figure 2A, Left). QTL on Chr 6 disappeared from replicate #4 when the number of strains was reduced to 27 (Figure 2A, Right). Thus, the QTL were also lost from the third replicate #5 when the number of strains was reduced to 23. In the last cycle of tests, when the number of strains was reduced to 21, three of five replicates failed to detect the QTL on Chr6. Although on average the detection probability of QTL at the suggestive level is 65%, the Pd of this QTL with strains of 20s is low.

For the QTL on Chr 1, after the number of strains was reduced to 35 or less, at least one of the five replicates failed to detect the QTL on Chr1 (Figure 2B, Right). Four of five replicates of 33 strains failed to reach a suggestive level (Figure 2B, Left). Thus, if an investigator uses 33 BXD strains or less, the Pd of a QTL at the significant level will be around 40%.

For the QTL on Chr13, the suggestive level was reached in all five replicates in the first cycle when the number of strains was 45 in five replicates. When the number of strains was reduced to 41, two replicates failed to reach the suggestive level (Figure 2C, Right). Three replicates of 39 strains failed to map to a significant level (Figure 2C, Left). Thus, with a number of strains less than 40, there is a lower Pd of QTL at suggestive level.

In addition to the loss of Pd in detection of real QTL, variations occur in the number of QTL (Figure 2D); major and minor QTL switch over (Figure 1B); and false positive loci appear (Figure 2D).

As the number of strains was reduced, the total numbers of QTL loci were also reduced. Figure 2D (Right) shows the number of QTL with LRS equal to or higher than the suggestive level. Furthermore, the rate of false positive loci increased while the number of strains was reduced. Figure 2D (Left) shows the percentage of false positive loci in each test cycle. This calculation assumes that the loci detected using the original 46 strains were the correct loci. The first false positive QTL on Chr17 reached the suggestive level in one of the five replicates when the number of strains was reduced to 41 (Figure 1B), while the QTL on Chr 1 had a lower LRS and QTL on Chr13 did not reach the suggestive level. After the number of strains was reduced to less than 39, at least one false positive QTL was detected as a major QTL in one of five replicates.

Most importantly, as the number of strains became less, the false positive loci became the major QTL. In addition to randomly appearing false positive loci, some false positive loci persisted and appeared as the major QTL in the same replicates. A typical example is the false positive locus on Chr17. It was initially detected in replicate #2 when the number of strains was 39 and less. It was consistently detected in the same replicate from all the tests of further reduced number of strains (Figure 1B).

**Figure 3:**
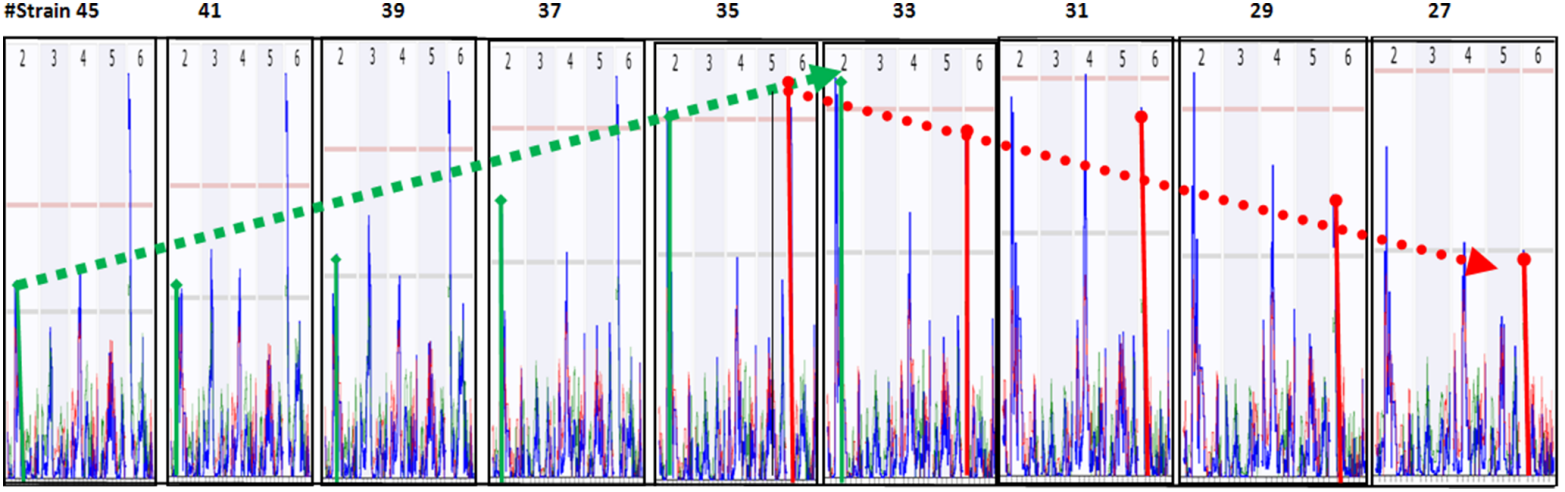
Switch over between major and minor QTL detected with different strains. The numbers on top of each figure indicate the number of chromosome. Numbers on left vertical bar indicate the LRS values. Figure 3A: QTL on Chr2 became one of the major QTL when test was conducted with 35 strains. Figure 3B: QTL on Chr2 became the major QTL with highest LRS score when the test was conducted with 33 strains. QTL on Chr2 became the major QTL with highest LRS score when the test was conducted with 31 strains. QTL on Chr2 became the only major QTL with highest LRS score when the test was conducted with 29 strains. QTL on Chr2 became the only detectable major QTL when the test was conducted with 27 strains.

There was a switch over between major and minor QTL. A QTL on Chr2 was among the four QTL with low LRS scores when the mapping was conducted with 46 original stains; however, it became the major QTL in replicate #1 when testing was conducted with 35 strains (Figure 3). When the number of strains was reduced to 33, it became one of the major QTL with the highest LRS score. When the number of strains was reduced to 29 and 27, it became the only major QTL.

### Number of strains required for accurate detection of QTL for cerebral cortex volume

The second set of data was from the cerebral cortex volume, adjusted for shrinkage, age, sex, plane of section, and BXD group/epoch [13]. The sample included a set of 56 BXD RI strains (GeneNetwork ID: 10997). Two QTL, one on chromosome Chr 6 at 88+/−5 Mb and the other at Chr 11 (41+/−8 Mb), have been identified.

**Figure 4:**
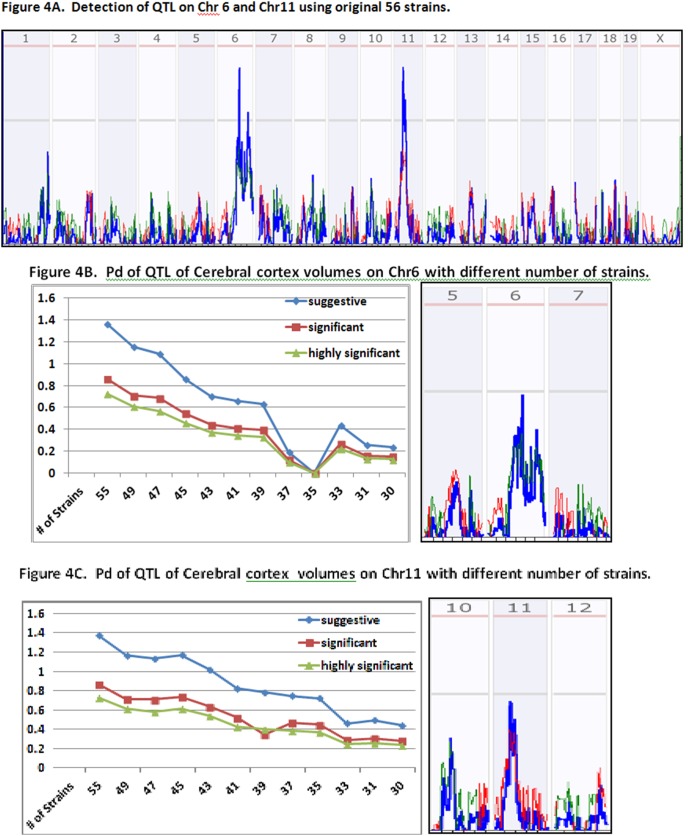
Pd of QTL of Cerebral cortex volumes with different number of strains. Y-axis indicates the Pd. X-axis indicates the number of RI strains in the test of Pd. Figure 4A: Detection of QTL on Chr 6 and Chr11 using original 56 strains. Figure 4B: Pd of QTL of Cerebral cortex volumes on Chr6 with different number of strains. Figure 4C: Pd of QTL of Cerebral cortex volumes on Chr11 with different number of strains.

We reproduced the reported QTL data on Chr6 and Chr11. With the 2000 permutation test, criteria for the mapping with the original data of 56 strains indicated a suggestive LRS of 10.09, significant LRS of 16.23, and highly significant LRS of 20.04. The data mapped the major QTL loci on Chr6 and Chr11 (Figure 4A).

The first locus was on Chr6, with an LRS score of 14.465. The second locus was on Chr11, with a LRS score of 14.394. Compared to QTL on Chr 6 of TNFa cytokine expression, these two QTL have relatively lower LRS scores and contributions to the phenotype. At the number of strains of 49, each of the five replicates produced the same QTL on both Chr 6 and Chr11 with a relatively lower Pd. The LRS of QTL on Chr 6 in one of the replicate did not reach the suggestive level for the first time when the number of strains was 46 (Figure 4B, Right). When the number of strains was reduced to 39, the Pd for the QTL on Chr 6 to be detected at suggestive level was reduced to 0.67 (Figure 4B, Left). After the number of strains was less than 37, there is only a Pd of less than 50% for this QTL. For the QTL on Chr11, it did not reach the suggestive level when the number of strains was 46 (Figure 4C, Right). The chance of 50% of Pd for the QTL is after the 35 strains (Figure 4C, Left). Therefore, after reducing the number of strains to less than 37, even if a correct QTL is detected, it will be only one of the two QTL, not the both.

**Figure 5:**
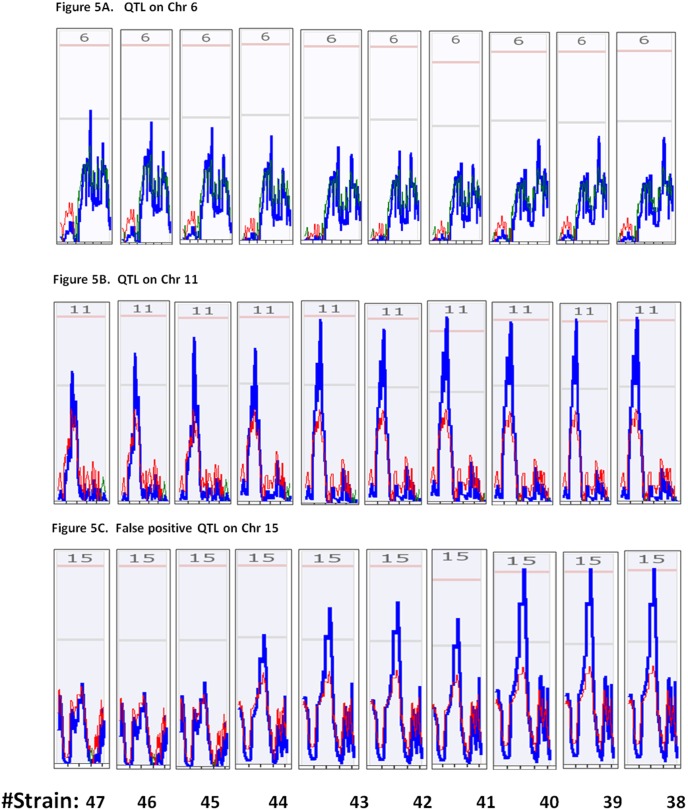
Persistence of false positive QTL on Chr 15 in the tests with less number of RI strains. Pink color lines on the top indicate the threshold for significant level. Light grey lines indicate the threshold for suggestive level. Top panel, detection of QTL on Chr 6 in the replicate #2 in the tests with different number of strains. Middle panel, detection of QTL on Chr 11 in the tests with different number of strains. Bottom panel, detection of false positive QTL on Chr 15 in the tests with different number of strains.

The false positive QTL on Chr 15 was first detected when the strains were reduced to 44 (Figure 5, Bottom). After that, at least one positive QTL was detected in every test of the five replicates (Figure 5). A persistent false positive QTL on Chr15 had the highest LRS score in one of five replicates with 40 RI strains (Figure 5). It was consistently detected in the same replicate from all the tests of further reduced number of strains. Figure 5 shows the detection of QTL of Chr 6, Chr 11 and false positive QTL on Chr 15 from the same replicate #2 when the number of strains was reduced to between 47 and 38. The QTL on Chr 6 was not detectable after number of strains was reduced to 46.

### Confirmation on number of strains requirement in three more traits

The GeneNetwork contains 3781 phenotype records, 1168 of which were uploaded before 2009. Most of them are with strains less than 30, and many have been published with genetic mapping as part of the results. We took data from an additional four sets that are published and have RI number of strains round 50 for further confirmation. For each of those sets, we sequentially reduced the number of strains to 35, 33, 31, and 29, with five replicates for each number of strains (Figure S1–S4).

Our results indicated that when the number of strains was reduced to the 30s, all three problems–rapid reductions of Pd, major-minor QTL switch over, and persistent appearance of false positive QTL as major locus–were dramatically amplified in all of four sets of data. Figure S1–S4 show examples of false positive loci in each of data set.

### Number of strains required for accurately detection of QTL from RI strains derived from a different parental pair and from rat

Above mentioned data were obtained from RI strains derived from the same pair of parents C57BL/6J (B6) X DBA/2J (D2). We further asked the question whether the limitation of strain numbers also occurred in RI strains derived from other pairs of mouse strains and from other mammalian model. We therefore tested the QTL mapping from RI strain derived from a reciprocal cross between A/J (A) and C57BL/6J [B] and from rat RI strains derived from a cross between the spontaneously hypertensive rat (SHR/OlaIpcv = H) and Brown Norway (BN.Lx/Cub or BN = B).

**Figure 6:**
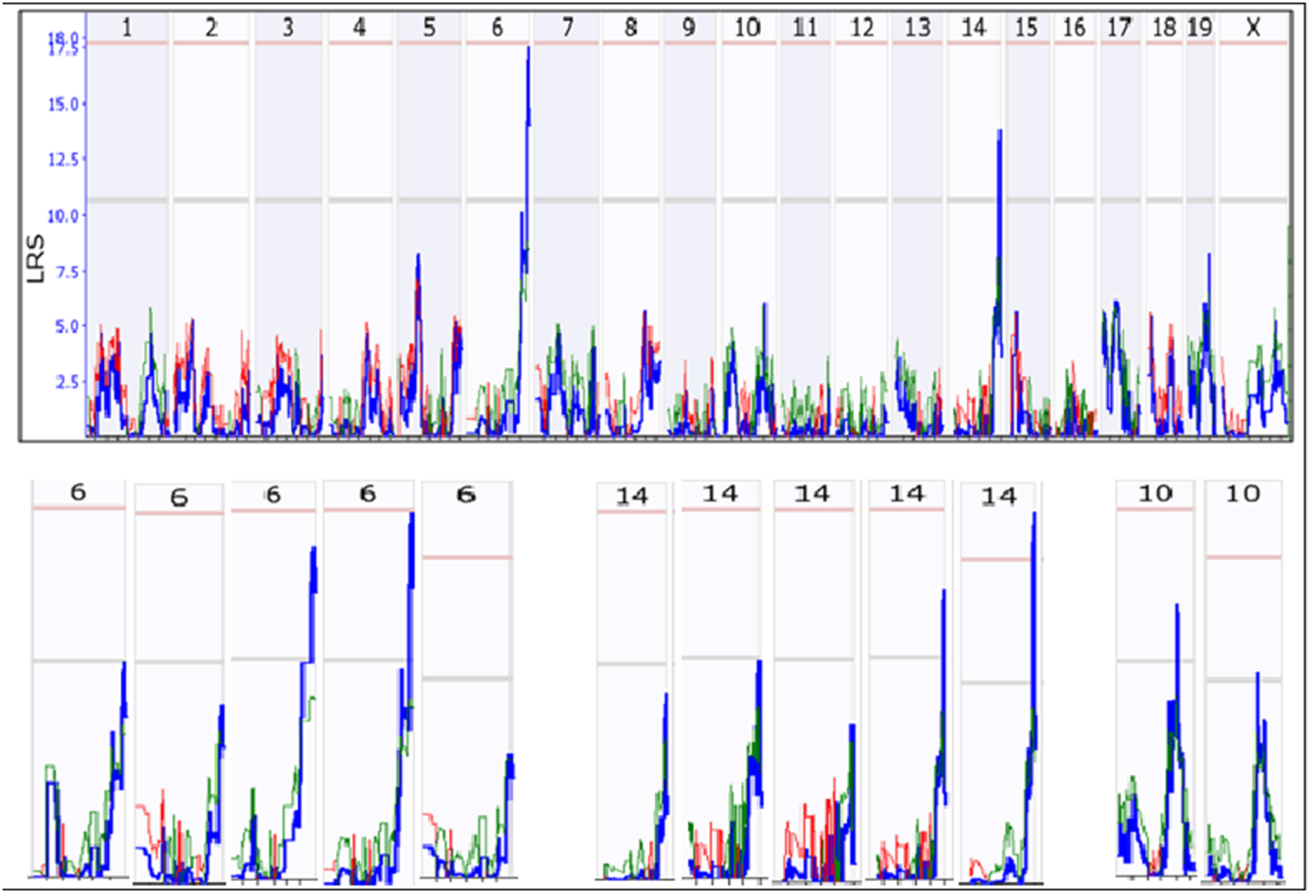
Failure detection of QTL with less number of RI strains derived from A/J and C57BL/6J. Upper panel shows the two QTL detected with the original 44 RI strains. Lower panel shows the frequency of detection of the two QTL when the number of RI strains was reduced to 30. The QTL on chromosome 6 and on chromosome 14 are both detected from two of the five replicates. A false positive QTL on chromosome 10 reached the suggestive level in two of the five replicates.

From the original AXB RI strains, two QTL loci were detected (Figure 6, upper panel). One is located on Chr 6 which reached a significant level. The other is on Chr14, which is at the level between suggestive and significant level. When the number of strains was reduced into 30, The QTL loci on both chromosomes were detected from two of the five replicates (Figure 6, lower panel). At the same time, a new or false QTL on Chr10 were detected in two of the five replicates.

**Figure 7:**
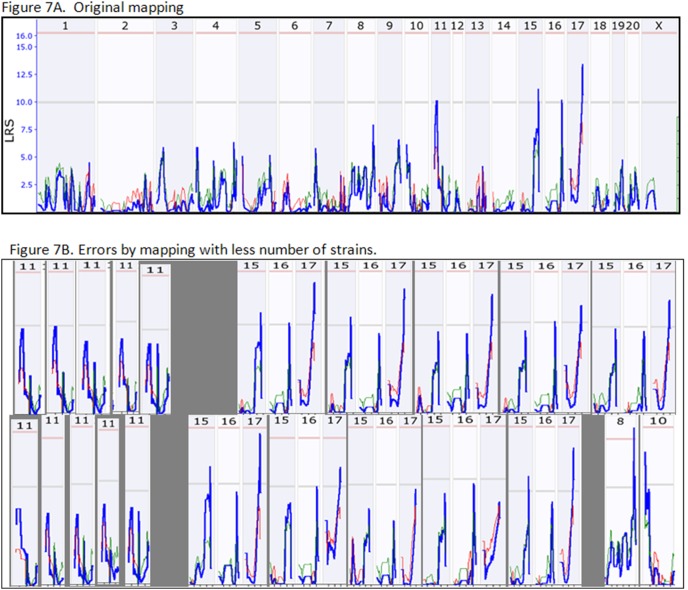
Reproducing ability of QTL in RI strains of rat. Figure 7A shows the QTL reached or above the suggestive levels. The upper panel in Figure 7B shows the QTL detected when two strains were eliminated from the RI strain population. The lower panel in Figure 7B shows the QTL detected when four RI strains were eliminated from the RI population.

In rat RI strains, four suggestive QTL were detected from the original strains. They are located on Chr 11, 15, 16, and 17 (Figure 7A). When we took two strains from the population and conducted QTL mapping, only the QTL on Chr17 was detected in each of five replicates (Figure 7B). QTL on Chr 15 and 16 reached suggestive levels in two out of five replicates. QTL on Chr11 did not reach suggestive in any replicates. When we took four strains out of the RI population, QTL on Chr 17 reached suggestive level in three of the five replicates (Figure 7B). QTL on Chr15 was detected at suggestive level from two of five replicates. QTL on Chr16 was detected from only one replicate and QTL on Chr 11 was not detected from any replicate. Most importantly, in one replicate, a false positive QTL on Chr 8 reached a significant level.

### Potential problems of reported QTL using small number of RI strains

Concerning the serious problem in QTL mapping with a small number of RI strains, we examined previous studies using relatively small numbers of RI strains in mice and rats. Using key words “recombinant inbred mapping QTL mouse” we found 215 publications. We went through the list and provisionally determined that at least 127 publications were based on the RI strains, most of which used RI strains of less than 40 (Table S1). In addition, at least 9 publications are conducted using the rat. Therefore, these publications should be regarded having a high risk of reporting false positive QTL, as well as missing important QTL. The QTL with a minor effect or low LRS/LOD scores from those studies should be considered unreliable.

## Discussion

Our data clarified the misconception that RI strains are mostly error-free in mapping and provided a partial answer to the puzzle as to why the causal genes for many QTL cannot be found and why some identified candidate genes cannot be confirmed. Because of the variation of crossover and genomic blocks between different strain pairs, the minimal number of RI strains needed for QTL mapping may vary in other RI populations. Limitations for the number of strains on the detection of QTL loci using other strategies and resources also should be evaluated to avoid similar situations as in RI strains.

The failure to detect the major QTL that is at the significant level from a small number of RI strains imposes a serious problem in the QTL mapping. In general, statistical methods for QTL detection include threshold of suggestive, significant, or highly significant. The permutation test in GeneNetwork is one method that is used to prevent and excessive number of false QTL discoveries in many QTL programs [18]. This threshold is computed by evaluating the distribution of highest LRS scores generated by a set of 2000 random permutations of strain means. When performing a single statistical test, GeneNetwork accepts a false discovery rate of 1 in 100 (p = 0.01) as a QTL at the highly significant level, 1 in 20 (p = 0.05) as a QTL at the significant level, and p = 0.63 as at the suggestive level (http://www.genenetwork.org/glossary.html). In our analysis, most tests produced multiple QTL loci. While the QTL at the suggestive levels are known prone to be false positive, major QTL in each test is near, at, or above the significant level. These major QTL, however, disappeared in about or more than 50% of the tests after the RI number of strains are reduced to around 30. Therefore, QTL mapping with a small number of RI strains has risk in not detecting the real major QTL.

The false positive QTL at the significant level detected from the small number of RI strains could mislead our scientific research and impose a long-term negative impact on our research. The detection of a false positive QTL at significant level has much negative impact than no detection of a QTL. The negative impact is a continued long-term effect and in the multiple levels. For example, such a QTL could mislead the researcher at the scientific filed to a continued effort to identify the underlined gene, to use it as the evidence of genetic effect on a trait, to cause confusion on the genetic mechanism of a trait. It does not only waste funding and effort, but also cheat the scientific world.

Understanding the limitation of RI number of strains on QTL identification allows investigators to identify what data on QTL and which populations are reliable to use at GeneNetwork and other databases. Before using the data, one would first have to carefully examine the number of RI strains used, the parental strains, and species for the mapping. If the number of strains seems small, further tests should be conducted before conducting further study on the QTL. As indicated in our data, not every QTL from a small number of RI strains is false positive. Because of the large number of QTL existing in the GeneNetwork and other databases, double checking with multiple resources will benefit in identification of a reliable QTL. For example, a QTL on mouse chromosome 2 for alcohol preference has been reported in GeneNetwork with a relatively small number of BXD RI strains [19], the same locus has also been reported from a previously study by Bennett et al [20]. Therefore, it most likely that the RI strain can be used to study the QTL for alcohol preference on chromosome 2.

Our analysis demonstrated that not only the standard inbred mouse strains, but also the RI strains have the limitation by its numbers. However, the limitations for the number on different traits and for different RI strains will vary because of the differences in homozygous genomic blocks in the RI populations.

One important note is that the minimum number of strains required in a QTL mapping seems varying in different RI strains. For example, it appears that minimum number of RI strains in rat in our analysis seems smaller than that in the RI strains in the mouse. In BXD RI mouse strains, when the number of strains is less than 40, suggestive QTL are not reliable, while the number of strains is less than 30, the QTL at significant level are not reliable. In rat, it appears that a reliable QTL can be detected at the number of strains around 30. The variation may be caused by the recombination frequency and block sizes of recombinant genome regions among RI strains. Thus, RI strains derived from same parents but developed at different time, from different parental strains, and from different species have different recombinant frequency and sizes of different genome blocks. Other factors such as data quality and data variations in phenotypes may also influence the ability of QTL mapping.

We noticed that recently Logan et al reported that they obtained high-precision QTL mapping results for 38 behavioral measures with 283 male and female of diversity outbred population mice [10]. Theoretically, outbred strains are more complex, and produce QTL with less accuracy. At present, there is not enough information to determine how precise these QTL are. However, we suspect that RI strains derived from diversity outbred populations also have the number limitations.

## Supporting Information

Figure S1 **Detection of QTL with original strains and false positive in reduced strain numbers in four sets of data from GeneNetwork.** Numbers on top of the figures are the chromosome numbers. Pink color lines indicate the threshold for significant level while the light grey lines for suggestive level. 1A. Detection of QTL on Chr 4 and Chr 17 in original 43 strains (GeneNetwork ID 12569) 1B. The detection of false positive QTL on Chr11 and none detectable QTL on Chr 4 and 17 when the strain number was reduced to 32.
(DOCX)

Figure S2 **Detection of QTL with original strains and false positive in reduced strain numbers in four sets of data from GeneNetwork.** Numbers on top of the figures are the chromosome numbers. Pink color lines indicate the threshold for significant level while the light grey lines for suggestive level. 2A. Detection of QTL on Chr 4 10 14 with original 46 strains (ID 12567) 2B. Detection of false positive QTL on Chr 5 and none detectable QTL on Chr 4 and 10 when the number of strains was reduced to 34.
(DOCX)

Figure S3 **Detection of QTL with original strains and false positive in reduced strain numbers in four sets of data from GeneNetwork.** Numbers on top of the figures are the chromosome numbers. Pink color lines indicate the threshold for significant level while the light grey lines for suggestive level. 3A. Detection of QTL on Chr 2 7, 11, 15, 17 with original 69 strains (ID 10866) 3B. Detection of false positive QTL on Chr 5 and none detectable QTL on Chr 7, 11 and 15 with 32 strains.
(DOCX)

Figure S4 **Detection of QTL with original strains and false positive in reduced strain numbers in four sets of data from GeneNetwork.** Numbers on top of the figures are the chromosome numbers. Pink color lines indicate the threshold for significant level while the light grey lines for suggestive level. 4A. Detection of QTL on Chr 4 9, and 16 with original 64 strains (ID 11485) 4B. Detection of false positive QTL on Chr 5, 11 and none detectable QTL on 9 with a less number of 30 strains.
(DOCX)

Table S1 **Publications of QTL mapping with RI strains.**
(DOCX)
