# Supplementary material for: Limitation of Number of Strains and Persistence of False Positive Loci in QTL Mapping Using Recombinant Inbred Strains
Source: PLoS One. 2014 Jul 17;9(7):e102307. doi: 10.1371/journal.pone.0102307 (PMC4102522; doi:10.1371/journal.pone.0102307)

Supplementary Figures. Detection of QTL with original strains and false positive in reduced strain numbers in four sets of data from GeneNetwork. Numbers on top of the figures are the chromosome numbers. Pink color lines indicate the threshold for significant level while the light grey lines for suggestive level.

**Figure S1.**

1A. Detection of QTL on Chr 4 and Chr 17 in original 43 strains (GeneNetwork ID 12569)


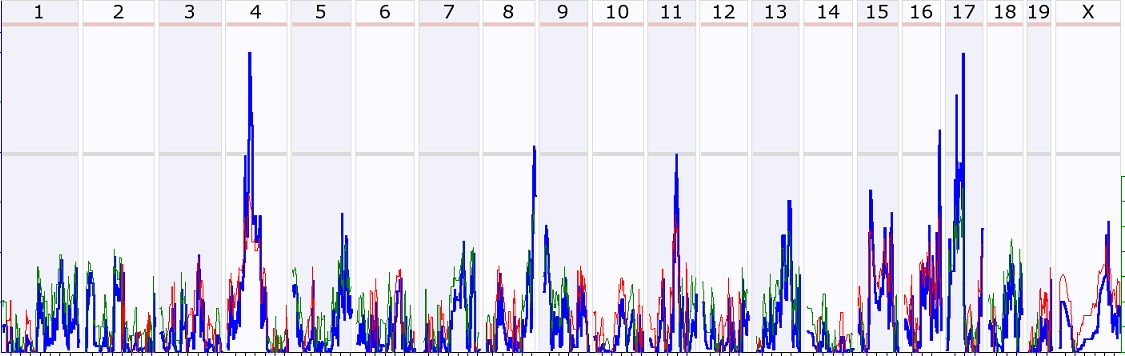


1B. The detection of false positive QTL on Chr11 and none detectable QTL on Chr 4 and 17 when the strain number was reduced to 32.


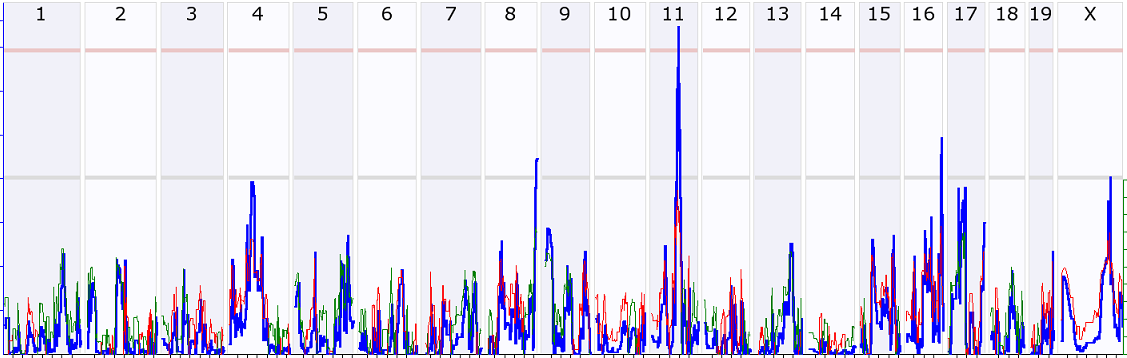

Supplement: Figure S1 — Detection of QTL with original strains and false positive in reduced strain numbers in four sets of data from GeneNetwork. Numbers on top of the figures are the chromosome numbers. Pink color lines indicate the threshold for significant level while the light grey lines for suggestive level. 1A. Detection of QTL on Chr 4 and Chr 17 in original 43 strains (GeneNetwork ID 12569) 1B. The detection of false positive QTL on Chr11 and none detectable QTL on Chr 4 and 17 when the strain number was reduced to 32. (DOCX) [file pone.0102307.s001.docx]
