# Supplementary material for: Limitation of Number of Strains and Persistence of False Positive Loci in QTL Mapping Using Recombinant Inbred Strains
Source: PLoS One. 2014 Jul 17;9(7):e102307. doi: 10.1371/journal.pone.0102307 (PMC4102522; doi:10.1371/journal.pone.0102307)

Supplementary Figures. Detection of QTL with original strains and false positive in reduced strain numbers in four sets of data from GeneNetwork. Numbers on top of the figures are the chromosome numbers. Pink color lines indicate the threshold for significant level while the light grey lines for suggestive level.

**Figure S2**.

2A. Detection of QTL on Chr 4 10 14 with original 46 strains (ID 12567)
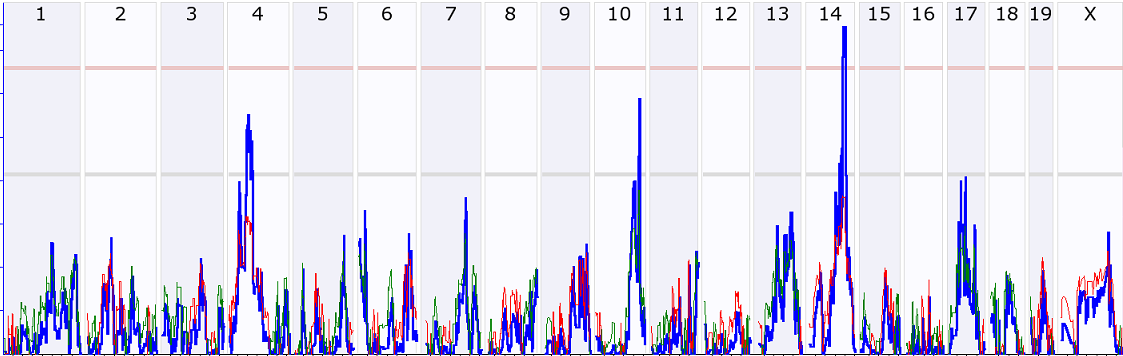


2B. Detection of false positive QTL on Chr 5 and none detectable QTL on Chr 4 and 10 when the number of strains was reduced to 34.


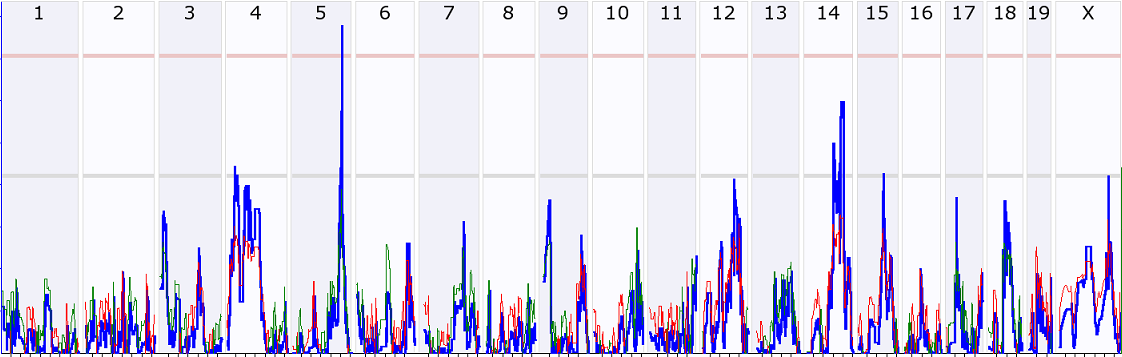

Supplement: Figure S2 — Detection of QTL with original strains and false positive in reduced strain numbers in four sets of data from GeneNetwork. Numbers on top of the figures are the chromosome numbers. Pink color lines indicate the threshold for significant level while the light grey lines for suggestive level. 2A. Detection of QTL on Chr 4 10 14 with original 46 strains (ID 12567) 2B. Detection of false positive QTL on Chr 5 and none detectable QTL on Chr 4 and 10 when the number of strains was reduced to 34. (DOCX) [file pone.0102307.s002.docx]
