# Supplementary material for: Limitation of Number of Strains and Persistence of False Positive Loci in QTL Mapping Using Recombinant Inbred Strains
Source: PLoS One. 2014 Jul 17;9(7):e102307. doi: 10.1371/journal.pone.0102307 (PMC4102522; doi:10.1371/journal.pone.0102307)

Supplementary Figures. Detection of QTL with original strains and false positive in reduced strain numbers in four sets of data from GeneNetwork. Numbers on top of the figures are the chromosome numbers. Pink color lines indicate the threshold for significant level while the light grey lines for suggestive level.

**Figure S3**.

3A. Detection of QTL on Chr 2 7, 11, 15, 17 with original 69 strains (ID 10866)
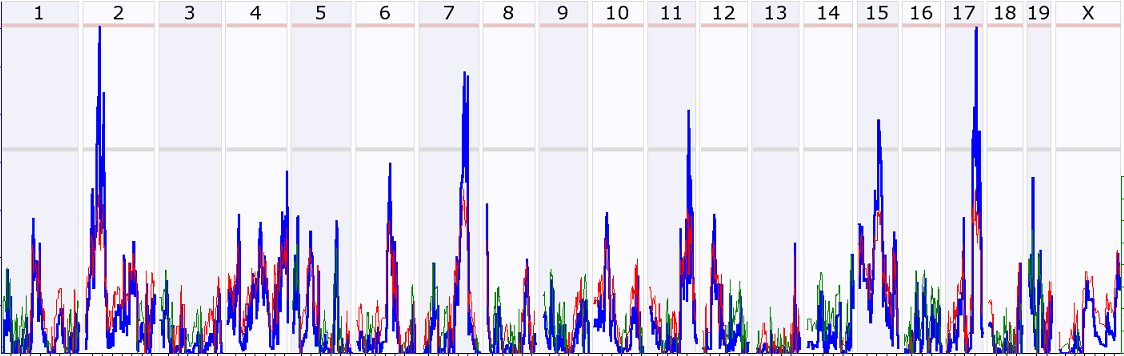


3B. Detection of false positive QTL on Chr 5 and none detectable QTL on Chr 7, 11 and 15 with 32 strains.


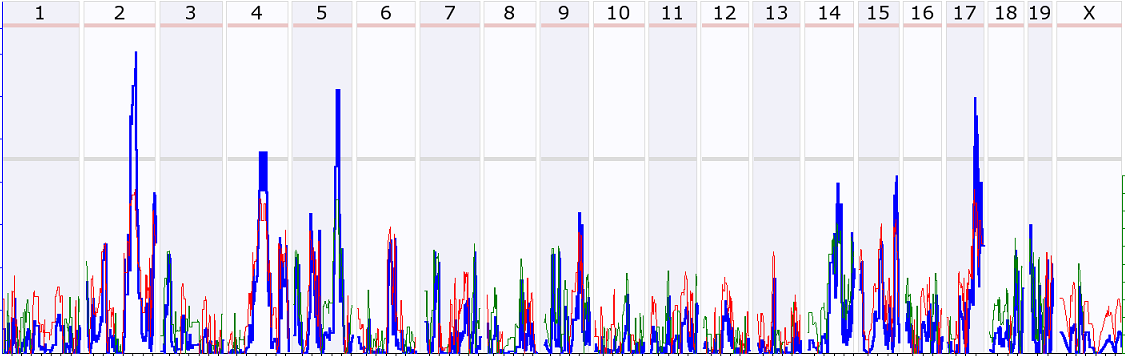

Supplement: Figure S3 — Detection of QTL with original strains and false positive in reduced strain numbers in four sets of data from GeneNetwork. Numbers on top of the figures are the chromosome numbers. Pink color lines indicate the threshold for significant level while the light grey lines for suggestive level. 3A. Detection of QTL on Chr 2 7, 11, 15, 17 with original 69 strains (ID 10866) 3B. Detection of false positive QTL on Chr 5 and none detectable QTL on Chr 7, 11 and 15 with 32 strains. (DOCX) [file pone.0102307.s003.docx]
