# Supplementary material for: Limitation of Number of Strains and Persistence of False Positive Loci in QTL Mapping Using Recombinant Inbred Strains
Source: PLoS One. 2014 Jul 17;9(7):e102307. doi: 10.1371/journal.pone.0102307 (PMC4102522; doi:10.1371/journal.pone.0102307)
Supplement: Table S1 — Publications of QTL mapping with RI strains. (DOCX) [file pone.0102307.s005.docx]

**Supplementary materials Table S1. Publications of QTL mapping with RI strains**

| **animal model** | **Title of the publication** | **strain number** | | **Year** | **publication information** |
| --- | --- | --- | --- | --- | --- |
| mouse | ATR-FTIR spectroscopy reveals genomic loci regulating the tissue response in high fat diet fed BXD recombinant inbred mouse strains. | 29 | | 2013 | BMC Genomics. 2013 Jun 10;14:386. doi: 10.1186/1471-2164-14-386. |
| mouse | Modeling host genetic regulation of influenza pathogenesis in the collaborative cross. | 8 strain and 155 pre-CC mice | | 2013 | PLoS Pathog. 2013 Feb;9(2):e1003196. doi: 10.1371/journal.ppat.1003196. Epub 2013 Feb 28. |
| mouse | Use of the expanded panel of BXD mice narrow QTL regions in ethanol-induced locomotor activation and motor incoordination. | 55 | | 2013 | Alcohol Clin Exp Res. 2013 Jan;37(1):170-83. doi: 10.1111/j.1530-0277.2012.01865.x. |
| mouse | Mapping of clinical and expression quantitative trait loci in a sex-dependent effect of host susceptibility to mouse-adapted influenza H3N2/HK/1/68. | 29 | | 2012 | J Immunol. 2012 Apr 15;188(8):3949-60. doi: 10.4049/jimmunol.1103320. Epub 2012 Mar 16. |
| mouse | Quantitative trait locus mapping for ethanol teratogenesis in BXD recombinant inbred mice. | 29 | | 2012 | Alcohol Clin Exp Res. 2012 Aug;36(8):1340-54. doi: 10.1111/j.1530-0277.2012.01754.x. Epub 2012 Mar 13. |
| mouse | Treatment- and population-dependent activity patterns of behavioral and expression QTLs. | multiple strains including 31 LXS strains, 30 and 69 BXD strains | | 2012 | PLoS One. 2012;7(2):e31805. doi: 10.1371/journal.pone.0031805. Epub 2012 Feb 16. |
| mouse | Expression QTL mapping in regulatory and helper T cells from the BXD family of strains reveals novel cell-specific genes, gene-gene interactions and candidate genes for auto-immune disease. | 31 | | 2011 | BMC Genomics. 2011 Dec 19;12:610. doi: 10.1186/1471-2164-12-610. |
| mouse | A QTL on chromosome 10 modulates cone photoreceptor number in the mouse retina. | 26 | | 2011 | Invest Ophthalmol Vis Sci. 2011 May 16;52(6):3228-36. doi: 10.1167/iovs.10-6693. |
| mouse | Genomic loci and candidate genes underlying inflammatory nociception. | 24 | | 2011 | Pain. 2011 Mar;152(3):599-606. doi: 10.1016/j.pain.2010.11.029. Epub 2010 Dec 30. |
| mouse | Appetitive operant conditioning in mice: heritability and dissociability of training stages. | 35 | | 2010 | Front Behav Neurosci. 2010 Nov 4;4:171. doi: 10.3389/fnbeh.2010.00171. |
| mouse | Quantitative trait loci analysis of tail tendon break time in mice of C57BL/6J and DBA/2J lineage. | 23 | | 2011 | Quantitative trait loci analysis of tail tendon break time in mice of C57BL/6J and DBA/2J lineage. |
| mouse | Positional cloning of a quantitative trait locus contributing to pain sensitivity: possible mediation by Tyrp1. | 35 | | 2010 | Genes Brain Behav. 2010 Nov;9(8):856-67. doi: 10.1111/j.1601-183X.2010.00618.x. |
| mouse | Genetic study of gutter-shaped root (GSR) in AKXL RI mouse strains using QTL analysis. | 7 strains 5 F1s | | 2010 | J Oral Sci. 2010 Jun;52(2):213-20. |
| mouse | Quantitative trait loci (QTL) analysis of longevity in C57BL/6J by DBA/2J (BXD) recombinant inbred mice. | 23 | | 2010 | Aging Clin Exp Res. 2010 Feb;22(1):8-19. |
| mouse | Identifying genetic loci and spleen gene coexpression networks underlying immunophenotypes in BXD recombinant inbred mice. | 41 | | 2010 | Physiol Genomics. 2010 Feb 23. [Epub ahead of print] |
| mouse | Genetic regulatory network analysis for app based on genetical genomics approach. | 78 | | 2010 | Exp Aging Res. 2010 Jan-Mar;36(1):79-93. doi: 10.1080/03610730903418729. |
| mouse | An experimental assessment of in silico haplotype association mapping in laboratory mice. | 81 | | 2009 | BMC Genet. 2009 Dec 9;10:81. doi: 10.1186/1471-2156-10-81. |
| mouse | Genetic architecture for hole-board behaviors across substantial time intervals in young, middle-aged and old mice. | 23 | | 2009 | Genes Brain Behav. 2009 Oct;8(7):714-27. doi: 10.1111/j.1601-183X.2009.00516.x. Epub 2009 Jun 23. |
| mouse | Replication and narrowing of gene expression quantitative trait loci using inbred mice. | 77 | | 2009 | Mamm Genome. 2009 Jul;20(7):437-46. doi: 10.1007/s00335-009-9199-0. Epub 2009 Jul 17. |
| mouse | The genetic control of neocortex volume and covariation with neocortical gene expression in mice. | 56 | | 2009 | BMC Neurosci. 2009 May 9;10:44. doi: 10.1186/1471-2202-10-44. |
| mouse | Expression quantitative trait loci and genetic regulatory network analysis reveals that Gabra2 is involved in stress responses in the mouse. | | | 2009 | Stress. 2009 Nov;12(6):499-506. doi: 10.3109/10253890802666112. |
| mouse | Genetic analysis of the psychostimulant effects of nicotine in chromosome substitution strains and F2 crosses derived from A/J and C57BL/6J progenitors. | | 21 | 2009 | Mamm Genome. 2009 Jan;20(1):34-42. doi: 10.1007/s00335-008-9159-0. Epub 2008 Dec 13. |
| mouse | Blood pressure and heart rate QTL in mice of the B6/D2 lineage: sex differences and environmental influences. | | 22 | 2009 | Physiol Genomics. 2009 Feb 2;36(3):158-66. doi: 10.1152/physiolgenomics.00035.2008. Epub 2008 Dec 9. |
| mouse | Chromosome 7q11 controls sperm beat cross frequency (BCF) in mice. | | - | 2004 | Folia Biol (Krakow). 2004;52(3-4):211-7. |
| mouse | Using gene expression databases for classical trait QTL candidate gene discovery in the BXD recombinant inbred genetic reference population: mouse forebrain weight. | | 34 | 2008 | BMC Genomics. 2008 Sep 25;9:444. doi: 10.1186/1471-2164-9-444. |
| mouse | The genetic contribution to heart rate and heart rate variability in quiescent mice. | | 59 | 2008 | Am J Physiol Heart Circ Physiol. 2008 Jul;295(1):H59-68. doi: 10.1152/ajpheart.00941.2007. Epub 2008 May 2. |
| mouse | Gene mapping of sperm quality parameters in recombinant inbred strains of mice. | | 12 | 2008 | Int J Dev Biol. 2008;52(2-3):287-93. doi: 10.1387/ijdb.072333ag. |
| mouse | Genetic regulation of hypothalamic cocaine and amphetamine-regulated transcript (CART) in BxD inbred mice. | | 26 | 2008 | Brain Res. 2008 Feb 15;1194:1-7. doi: 10.1016/j.brainres.2007.11.074. Epub 2007 Dec 14. |
| mouse | Genetic analysis reveals polygenic influences on iron, copper, and zinc in mouse hippocampus with neurobiological implications. | | 30 | 2008 | Hippocampus. 2008;18(4):398-410. doi: 10.1002/hipo.20399. |
| mouse | Genetic analysis of posterior medial barrel subfield (PMBSF) size in somatosensory cortex (SI) in recombinant inbred strains of mice. | | 42 | 2008 | BMC Neurosci. 2008 Jan 7;9:3. doi: 10.1186/1471-2202-9-3. |
| mouse | Interspecific recombinant congenic strains between C57BL/6 and mice of the Mus spretus species: a powerful tool to dissect genetic control of complex traits. | | 55 | 2007 | Genetics. 2007 Dec;177(4):2321-33. Epub 2007 Oct 18. |
| mouse | Quantitative trait locus (QTL) mapping in aging systems. | | - | 2007 | Methods Mol Biol. 2007;371:321-48. |
| mouse | Genome-level analysis of genetic regulation of liver gene expression networks. | | 37 | 2007 | Hepatology. 2007 Aug;46(2):548-57. |
| mouse | The AcB/BcA recombinant congenic strains of mice: strategies for phenotype dissection, mapping and cloning of quantitative trait genes. | | 36 | 2007 | Novartis Found Symp. 2007;281:141-53; discussion 153-5, 208-9. |
| mouse | An integrative genomics strategy for systematic characterization of genetic loci modulating phenotypes. | | 42 | 2007 | Hum Mol Genet. 2007 Jun 1;16(11):1381-90. Epub 2007 Apr 11. |
| mouse | Quantitative trait loci linked to thalamus and cortex gray matter volumes in BXD recombinant inbred mice. | | 36 | 2007 | Heredity (Edinb). 2007 Jul;99(1):62-9. Epub 2007 Apr 4. |
| mouse | Principal component analysis of quantitative trait loci for immune response to adenovirus in mice. | | 18 | 2006 | Hereditas. 2006 Dec;143(2006):189-97. |
| mouse | Quantitative trait locus mapping for acute functional tolerance to ethanol in the L x S recombinant inbred panel. | | 74 | 2007 | Alcohol Clin Exp Res. 2007 Feb;31(2):200-8. |
| mouse | Genetic and structural analysis of the basolateral amygdala complex in BXD recombinant inbred mice. | | 35 | 2007 | Behav Genet. 2007 Jan;37(1):223-43. Epub 2006 Nov 28. |
| mouse | Independent quantitative trait loci influence ventral and dorsal hippocampal volume in recombinant inbred strains of mice. | | 36 BXD, 30 AXB/BXA | 2006 | Genes Brain Behav. 2006 Nov;5(8):614-23. |
| mouse | QTL analysis of trabecular bone in BXD F2 and RI mice. | | 23 | 2006 | J Bone Miner Res. 2006 Aug;21(8):1267-75. |
| mouse | Confirmation and fine mapping of ethanol sensitivity quantitative trait loci, and candidate gene testing in the LXS recombinant inbred mice. | | 75 | 2006 | J Pharmacol Exp Ther. 2006 Oct;319(1):299-307. Epub 2006 Jun 27. |
| mouse | QTL mapping for low-dose ethanol activation in the LXS recombinant inbred strains. | | 75 | 2006 | Alcohol Clin Exp Res. 2006 Jul;30(7):1111-20. |
| mouse | Identification of informative strains and provisional QTL mapping of amphetamine (AMPH)-induced locomotion in recombinant congenic strains (RCS) of mice. | | 34 | 2006 | Behav Genet. 2006 Nov;36(6):903-13. Epub 2006 May 19. |
| mouse | Genomic regulation of natural variation in cortical and noncortical brain volume. | | 34 | 2006 | BMC Neurosci. 2006 Feb 17;7:16. |
| mouse | Integrative genetic analysis of transcription modules: towards filling the gap between genetic loci and inherited traits. | | 32 | 2006 | Hum Mol Genet. 2006 Feb 1;15(3):481-92. Epub 2005 Dec 21. |
| mouse | Genetics of body weight in the LXS recombinant inbred mouse strains. | | 75 | 2005 | Mamm Genome. 2005 Oct;16(10):764-74. Epub 2005 Oct 29. |
| mouse | Genetic analysis of the hypothalamic neurotensin system. | | 26 | 2006 | Neuropsychopharmacology. 2006 Mar;31(3):535-43. |
| mouse | Genetic analysis of alcohol intake in recombinant inbred and congenic strains derived from A/J and C57BL/6J progenitors. | | 27 | 2005 | Mamm Genome. 2005 May;16(5):319-31. |
| mouse | Genetic regulation of bone mineral density in mice. | | 24 | 2002 | J Musculoskelet Neuronal Interact. 2002 Mar;2(3):232-6. |
| mouse | Uncovering regulatory pathways that affect hematopoietic stem cell function using 'genetical genomics'. | | 30 | 2005 | Nat Genet. 2005 Mar;37(3):225-32. Epub 2005 Feb 13. |
| mouse | Genetic analysis of the hypothalamic corticotropin-releasing factor system. | | 28 | 2005 | Endocrinology. 2005 May;146(5):2362-8. Epub 2005 Feb 10. |
| mouse | Interacting genetic loci cause airway hyperresponsiveness. | | - | 2005 | Physiol Genomics. 2005 Mar 21;21(1):105-11. Epub 2005 Jan 18. |
| mouse | Quantitative trait loci analysis of structural and material skeletal phenotypes in C57BL/6J and DBA/2 second-generation and recombinant inbred mice. | | 23 | 2005 | J Bone Miner Res. 2005 Jan;20(1):88-99. Epub 2004 Oct 11 |
| mouse | Genetic regulation of thymic involution. | | 20 | 2005 | Mech Ageing Dev. 2005 Jan;126(1):87-97. |
| mouse | Quantitative trait loci for obesity- and diabetes-related traits and their dietary responses to high-fat feeding in LGXSM recombinant inbred mouse strains. | | 16 | 2004 | Diabetes. 2004 Dec;53(12):3328-36. |
| mouse | Genetic regulation of endotoxin-induced airway disease. | | 34 | 2004 | Genomics. 2004 Jun;83(6):961-9. |
| mouse | A new set of BXD recombinant inbred lines from advanced intercross populations in mice. | | 46 | 2004 | BMC Genet. 2004 Apr 29;5:7. |
| mouse | Quantitative trait Loci specifying the response of body temperature to dietary restriction. | | 22 | 2004 | J Gerontol A Biol Sci Med Sci. 2004 Feb;59(2):118-25. |
| mouse | Genetic loci that control vascular endothelial growth factor-induced angiogenesis. | | 28 | 2003 | FASEB J. 2003 Nov;17(14):2112-4. Epub 2003 Sep 4. |
| mouse | Age-related thymic involution in C57BL/6J x DBA/2J recombinant-inbred mice maps to mouse chromosomes 9 and 10. | | 21 | 2003 | Genes Immun. 2003 Sep;4(6):402-10 |
| mouse | Mapping quantitative trait loci mediating sensitivity to etomidate. | | - | 2003 | Mamm Genome. 2003 Jun;14(6):367-75. |
| mouse | Confirmation of quantitative trait loci for cocaine-induced activation in the AcB/BcA series of recombinant congenic strains. | | 37 | 2003 | Pharmacogenetics. 2003 Jun;13(6):329-38. |
| mouse | Genetic analyses of ethanol-induced hyperglycemia. | | 21 | 2003 | Alcohol Clin Exp Res. 2003 May;27(5):756-64. |
| mouse | Quantitative trait loci modulate ventricular size in the mouse brain. | | 26 | 2003 | J Comp Neurol. 2003 Jun 30;461(3):362-9. |
| mouse | Quantitative trait loci on chromosomes 10 and 11 influencing mandible size of SMXA RI mouse strains. | | 23 | 2002 | J Dent Res. 2002 Jul;81(7):501-4. |
| mouse | Mapping of quantitative trait loci underlying ethanol metabolism in BXD recombinant inbred mouse strains. | | 25 | 2002 | Alcohol Clin Exp Res. 2002 May;26(5):610-6. |
| mouse | Parental genetic contributions in the AXB and BXA recombinant inbred mouse strains. | | - | 2002 | Mamm Genome. 2002 Mar;13(3):127-33. |
| mouse | Quantitative trait locus mapping of susceptibilities to butylated hydroxytoluene-induced lung tumor promotion and pulmonary inflammation in CXB mice. | | 13 | 2002 | Carcinogenesis. 2002 Mar;23(3):411-7. |
| mouse | Gene coding variant in Cas1 between the C57BL/6J and DBA/2J inbred mouse strains: linkage to a QTL for ethanol-induced locomotor activation. | | 36 | 2002 | Alcohol Clin Exp Res. 2002 Jan;26(1):1-7. |
| mouse | Quantitative trait loci regulating relative lymphocyte proportions in mouse peripheral blood. | | 35 | 2002 | Blood. 2002 Jan 15;99(2):561-6. |
| mouse | The genetic structure of recombinant inbred mice: high-resolution consensus maps for complex trait analysis. | | 100 | 2001 | Genome Biol. 2001;2(11):RESEARCH0046. Epub 2001 Oct 22. |
| mouse | Confirmation and fine mapping of chromosomal regions influencing peak bone mass in mice. | | 24 | 2001 | J Bone Miner Res. 2001 Nov;16(11):1953-61. |
| mouse | Further characterization and high-resolution mapping of quantitative trait loci for ethanol-induced locomotor activity. | | 25 | 2001 | Behav Genet. 2001 Jan;31(1):79-91. |
| mouse | Mapping quantitative trait loci for circadian behavioral rhythms in SMXA recombinant inbred strains. | | - | 2000 | Behav Genet. 2000 Nov;30(6):447-53. |
| mouse | Identification of quantitative trait loci controlling activation of TRBV4 CD8+ T cells during murine gamma-herpesvirus-induced infectious mononucleosis. | | - | 2001 | Immunogenetics. 2001 Jul;53(5):395-400. |
| mouse | Mapping genes that regulate density of dopamine transporters and correlated behaviors in recombinant inbred mice. | | 21 | 2001 | J Pharmacol Exp Ther. 2001 Aug;298(2):634-43. |
| mouse | Sensitivity of AXB/BXA recombinant inbred lines of mice to the locomotor activating effects of cocaine: a quantitative trait loci analysis. | | - | 2001 | Pharmacogenetics. 2001 Apr;11(3):255-64. |
| mouse | The homeostatic regulation of sleep need is under genetic control. | | 25 | 2001 | J Neurosci. 2001 Apr 15;21(8):2610-21. |
| mouse | Distribution of body weight, blood insulin and lipid levels in the SMXA recombinant inbred strains and the QTL analysis. | | 23 | 2000 | Exp Anim. 2000 Jul;49(3):217-24. |
| mouse | Scanning of five chromosomes for alcohol consumption loci. | | 80 | 2000 | Alcohol. 2000 Aug;22(1):25-34. |
| mouse | Identification and time dependence of quantitative trait loci for basal locomotor activity in the BXD recombinant inbred series and a B6D2 F2 intercross. | | 25 | 2000 | Behav Genet. 2000 May;30(3):159-70. |
| mouse | Mapping quantitative trait loci that regulate sensitivity and tolerance to quinpirole, a dopamine mimetic selective for D(2)/D(3) receptors. | | 24 | 2000 | Am J Med Genet. 2000 Oct 9;96(5):696-705. |
| mouse | Allelic variation in the GABA A receptor gamma2 subunit is associated with genetic susceptibility to ethanol-induced motor incoordination and hypothermia, conditioned taste aversion, and withdrawal in BXD/Ty recombinant inbred mice. | | 26 | 2000 | Alcohol Clin Exp Res. 2000 Sep;24(9):1327-34. |
| mouse | Quantitative trait locus mapping of genes regulating pulmonary PKC activity and PKC-alpha content. | | 22 | 2000 | Am J Physiol Lung Cell Mol Physiol. 2000 Aug;279(2):L326-32. |
| mouse | Genetic susceptibility to ozone-induced lung hyperpermeability: role of toll-like receptor 4. | | - | 2000 | Am J Respir Cell Mol Biol. 2000 May;22(5):620-7. |
| mouse | A quantitative genetic analysis of slow-wave sleep in influenza-infected CXB recombinant inbred mice. | | 13 | 1999 | Behav Genet. 1999 Sep;29(5):339-48. |
| mouse | Quantitative trait loci affecting ethanol sensitivity in BXD recombinant inbred mice. | | 25 | 2000 | Alcohol Clin Exp Res. 2000 Jan;24(1):17-23. |
| mouse | Quantitative-trait loci analysis of cocaine-related behaviours and neurochemistry. | | - | 1999 | Pharmacogenetics. 1999 Oct;9(5):607-17. |
| mouse | Provisional QTL for circadian period of wheel running in laboratory mice: quantitative genetics of period in RI mice. | | - | 1999 | Chronobiol Int. 1999 May;16(3):269-79. |
| mouse | Identification of an acute ethanol response quantitative trait locus on mouse chromosome 2. | | 25 | 1999 | J Neurosci. 1999 Jan 15;19(2):549-61. |
| mouse | Genetic association of a GABA(A) receptor gamma2 subunit variant with severity of acute physiological dependence on alcohol. | | - | 1998 | Mamm Genome. 1998 Dec;9(12):975-8. |
| mouse | Genes on mouse chromosomes 2 and 9 determine variation in ethanol consumption. | | - | 1998 | Mamm Genome. 1998 Dec;9(12):936-41. |
| mouse | Alcohol preference in AXB/BXA recombinant inbred mice: gender differences and gender-specific quantitative trait loci. | | - | 1998 | Mamm Genome. 1998 Dec;9(12):929-35. |
| mouse | Ethanol-induced conditioned taste aversion in BXD recombinant inbred mice. | | 20 | 1998 | Alcohol Clin Exp Res. 1998 Sep;22(6):1234-44. |
| mouse | Provisional quantitative trait loci (QTL) for the Aschoff effect in RI mice. | | - | 1998 | Physiol Behav. 1998 Apr;64(1):97-101. |
| mouse | Provisional mapping of quantitative trait loci for chronic ethanol withdrawal severity in BXD recombinant inbred mice. | | 25 | 1998 | J Pharmacol Exp Ther. 1998 Jul;286(1):263-71. |
| mouse | Effect of within-strain sample size on QTL detection and mapping using recombinant inbred mouse strains. | | 25 | 1998 | Behav Genet. 1998 Jan;28(1):29-38. |
| mouse | Localization of genes mediating acute and sensitized locomotor responses to cocaine in BXD/Ty recombinant inbred mice. | | 25 | 1998 | J Neurosci. 1998 Apr 15;18(8):3023-34. |
| mouse | Identification of a genetic region in mice that specifies sensitivity to propofol. | | 24 | 1998 | Anesthesiology. 1998 Feb;88(2):379-89 |
| mouse | Natural variation in neuron number in mice is linked to a major quantitative trait locus on Chr 11. | | 38 | 1998 | J Neurosci. 1998 Jan 1;18(1):138-46. |
| mouse | Localization of candidate genomic regions influencing paradoxical sleep in mice. | | - | 1997 | Neuroreport. 1997 Dec 1;8(17):3755-8. |
| mouse | Identification of a sex-specific quantitative trait locus mediating nonopioid stress-induced analgesia in female mice. | | 27 | 1997 | J Neurosci. 1997 Oct 15;17(20):7995-8002. |
| mouse | Brain dopamine receptor plasticity: testing a diathesis-stress hypothesis in an animal model. | | - | 1997 | Psychopharmacology (Berl). 1997 Jul;132(2):153-60. |
| mouse | Identification of quantitative trait loci involved in contextual and auditory-cued fear conditioning in BXD recombinant inbred strains. | | - | 1997 | Behav Neurosci. 1997 Apr;111(2):292-300. |
| mouse | Common quantitative trait loci for alcohol-related behaviors and central nervous system neurotensin measures: hypnotic and hypothermic effects. | | 24 | 1997 | J Pharmacol Exp Ther. 1997 Feb;280(2):911-8. |
| mouse | Quantitative trait loci affecting methamphetamine responses in BXD recombinant inbred mouse strains. | | 25 | 1997 | J Neurosci. 1997 Jan 15;17(2):745-54. |
| mouse | Quantitative trait loci contributing to phencyclidine-induced and amphetamine-induced locomotor behavior in inbred mice. | | 26 | 1996 | Neuropsychopharmacology. 1996 Nov;15(5):484-90. |
| mouse | Hypothetical quantitative trait loci (QTL) for circadian period of locomotor activity in CXB recombinant inbred strains of mice. | | 13 | 1996 | Behav Genet. 1996 Sep;26(5):505-11. |
| mouse | Quantitative trait loci for ethanol sensitivity in the LS x SS recombinant inbred strains: interval mapping. | | - | 1996 | Behav Genet. 1996 Jul;26(4):447-58. |
| mouse | Quantitative trait locus analyses of sleep-times induced by sedative-hypnotics in LSXSS recombinant inbred strains of mice. | | 28 | 1996 | Alcohol Clin Exp Res. 1996 May;20(3):543-50. |
| mouse | Mapping the genes for haloperidol-induced catalepsy. | | 26 | 1996 | J Pharmacol Exp Ther. 1996 May;277(2):1016-25. |
| mouse | Type I and type II error rates for quantitative trait loci (QTL) mapping studies using recombinant inbred mouse strains. | | 26 | 1996 | Behav Genet. 1996 Mar;26(2):149-60. |
| mouse | Genetic analysis of the corticosterone response to ethanol in BXD recombinant inbred mice. | | - | 1995 | Behav Neurosci. 1995 Dec;109(6):1199-208. |
| mouse | Chromosomal mapping of the psychomotor stimulant effects of cocaine in BXD recombinant inbred mice. | | 11 | 1995 | Psychopharmacology (Berl). 1995 Dec;122(3):209-14. |
| mouse | Quantitative trait loci (QTL) for circadian rhythms of locomotor activity in mice. | | 29 | 1995 | Behav Genet. 1995 Nov;25(6):545-56. |
| mouse | Quantitative trait loci associated with the behavioral response of B x D recombinant inbred mice to restraint stress: a preliminary communication. | | 24 | 1995 | Behav Genet. 1995 Sep;25(5):489-95. |
| mouse | Identification of a locus on mouse chromosome 17 associated with high-affinity choline uptake using BXD recombinant inbred mice and quantitative trait loci analysis. | | 27 | 1995 | Genomics. 1995 May 1;27(1):161-4. |
| mouse | Alcohol acceptance, preference, and sensitivity in mice. II. Quantitative trait loci mapping analysis using BXD recombinant inbred strains. | | 27 | 1995 | Alcohol Clin Exp Res. 1995 Apr;19(2):367-73. |
| mouse | Effects of acute and repeated ethanol exposures on the locomotor activity of BXD recombinant inbred mice. | | 23 | 1995 | Alcohol Clin Exp Res. 1995 Apr;19(2):269-78. |
| mouse | Chromosomal mapping of loci influencing sensitivity to cocaine-induced seizures in BXD recombinant inbred strains of mice. | | 26 | 1995 | Psychopharmacology (Berl). 1995 Jan;117(1):62-6. |
| mouse | Localization of genes affecting alcohol drinking in mice. | | 19 | 1994 | Alcohol Clin Exp Res. 1994 Aug;18(4):931-41. |
| mouse | Quantitative trait loci mapping of genes that influence the sensitivity and tolerance to ethanol-induced hypothermia in BXD recombinant inbred mice. | | 19 | 1994 | J Pharmacol Exp Ther. 1994 Apr;269(1):184-92. |
| mouse | Mapping quantitative trait loci for ethanol-induced anesthesia in LSxSS recombinant inbred and F2 mice: methodology and results. | | 29 | 1994 | Alcohol Alcohol Suppl. 1994;2:79-86. |
| mouse | Use of recombinant inbred strains for studying genetic determinants of responses to alcohol. | | 21 | 1994 | Alcohol Alcohol Suppl. 1994;2:67-71. |
| mouse | Quantitative trait loci associated with brain weight in the BXD/Ty recombinant inbred mouse strains. | | 20 | 1992 | Brain Res Bull. 1992 Sep-Oct;29(3-4):337-44. |
| mouse | Single-locus control of saccharin intake in BXD/Ty recombinant inbred (RI) mice: some methodological implications for RI strain analysis. | | 20 | 1992 | Behav Genet. 1992 Jan;22(1):81-100. |
| Rat | Quantitative trait loci determining weight reduction of testes and pituitary by diethylstilbesterol in LEXF and FXLE recombinant inbred strain rats. | | 21+7 | 2006 | Exp Anim. 2006 Apr;55(2):91-5. |
| Rat | Identification of quantitative trait Loci for anxiety and locomotion phenotypes in rat recombinant inbred strains. | | 22 | 2004 | Behav Genet. 2004 Jan;34(1):93-103. |
| Rat | Genome scanning of the HXB/BXH sets of recombinant inbred strains of the rat for quantitative trait loci associated with conditioned taste aversion. | | 30 | 2002 | Behav Genet. 2002 Jan;32(1):51-6. |
| Rat | Identification and chromosomal localization of ecogenetic components of electrolyte excretion. | | 26 | 2002 | J Hypertens. 2002 Feb;20(2):209-17. |
| Rat | Chromosomal mapping of a major quantitative trait locus regulating compensatory renal growth in the rat. | | 29 | 2000 | J Am Soc Nephrol. 2000 Jul;11(7):1261-5. |
| Rat | Mapping of quantitative trait loci (QTL) of differential stress gene expression in rat recombinant inbred strains. | | 20 | 2000 | J Hypertens. 2000 May;18(5):545-51. |
| Rat | Linkage mapping of the Fos cellular oncogene (Fos) to rat chromosome 6 and its possible role in the regulation of compensatory renal growth. | | 30 | 1998 | Folia Biol (Praha). 1998;44(5):151-3. |
| Rat | Mapping of quantitative trait loci for seminal vesicle mass and litter size to rat chromosome 8. | | 30 | 1999 | J Reprod Fertil. 1999 Jul;116(2):329-33. |
| Rat | A genetic and correlation analysis of liver cholesterol concentration in rat recombinant inbred strains fed a high cholesterol diet. | | 30 | 1998 | Biochem Biophys Res Commun. 1998 May 8;246(1):272-5. |
